# On the Austral-Antarctic stenothoids *Proboloides*, *Metopoides*, *Torometopa* and *Scaphodactylus* (Crustacea Amphipoda) Part 2: the genus *Proboloides*, with description of two new genera and the transfer of two nominal species to *Metopoides*

**DOI:** 10.3897/zookeys.86.785

**Published:** 2011-03-19

**Authors:** Traudl Krapp-Schickel

**Affiliations:** Department of Biology, University of Montenegro, Cetinjski put b.b., 81000 Podgorica, Serbia and Montenegro

**Keywords:** Stenothoidae sensu lato, systematics, phylogenetic analysis, gen. *Proboloides*, *Malvinometopa* gen. n., *Victometopa* gen. n., *Victometopa rorida* sp. n.

## Abstract

This is the second part of a revision of the most plesiomorphic genera in the amphipod family Stenothoidae sensu lato (see Krapp-Schickel and Koenemann 2006 for an overview and Krapp-Schickel 2008 for the first part). 41 species not belonging to *Metopoides* were plotted in a matrix using the same 61 characters as in the first part. The resulting group of *Proboloides* species (most probably not existing in the Austral-Antarctic region) is discussed, a key for the members given and two new genera erected. Some species described as *Proboloides* are redescribed and 2 species transferred to *Metopoides*. A key for all actual members of. The remaining species, i.e. those actually being in the genera *Torometopa* and *Scaphodactylus*, will be dealt with in the final part of this series, together with a key to all of them.

## Introduction

Barnard and Karaman (1991: 694 ff.) list 23 species belonging to the genera *Metopoides* and *Proboloides*, which were always difficult to differenciate. Since this publication the number of species has increased, while our knowledge on character states did not grow the same way. The results of a phylogenetic analysis of the entire family Stenothoidae sensu lato by Krapp-Schickel and Koenemann (2006) showed not only that the genera treated therein had many plesiomorphic character states, but also that too many characters were still unknown or poorly described. Thus before further studies of the phylogenetic relationships, several species required redescription or at least checking of new characters not described so far.

## Material and methods

As many species as possible of this group were studied and redescribed, in order to replace the (initially numerous) question marks in the start-up matrix. Species were borrowed from different Museums.

**Acronyms for Museums**

AMS	Australian Museum Sydney

BMNH	British Museum (Natural History), London

MNVCr	Museo civico di Storia Naturale Verona

NMV	Museum of Victoria, Melbourne, Australia

ZMUC	Zoological Museum, University of Copenhagen or Købnhavn

**Abbreviations in taxonomical descriptions as well as figures**

A1, 2	antenna 1, 2

acc.	accessory

art	article

Cx	coxal plate

Ep	epimeral plate

flag	flagellum

Gn1, 2	gnathopod 1, 2

IP	inner plate

LL	lower lip

Md	mandible

Mx1, 2	maxilla 1, 2

Mxp	maxilliped

OP	outer plate

P3-7	peraeopod 3–7

ped	peduncle

T	telson

U1–3	uropod 1–3

UL	upper lip

Us	urosome

## Character matrix

The chosen characters were as follows:

Head

**(1.)** *A1 length* (0) > A2; (1) ≤ A2

**(2.)** *A2 peduncle article 1 ratio length: breadth* (0) ≤ 3; (1) > 3

**(3.)** *ratio A1: body length* (0) ≥ 0.66% body; (1) < 0.66% body

**(4.)** *A1 flagellum acc.* (0) many articles; (1) 2–1 articles; ; (2) lacking

**(5.)** *A1 peduncle art 2*  (0) < article 1; (1) ≥ article 1

**(6.)** *A1 peduncle art 1* (0) < ceph.; (1) = ceph.; (2) > ceph.

**(7.)** *A1 peduncle art 3* (0) ≤0.3 art 1 ; (1) 0.3–0.5 art 1; (2) ≥ 0.5 art 1

**(8.)** *A1 flagellum arts* (0) <10; (1) 11–20; (2) 21–30; (3) >30

**(9.)** *A2 peduncle art 5* (0) < flag,; (1) = flag.; (2) > flag.

**(10.)** *A2 peduncle art 4* (0) >art 5; (1) =art 5; (2) <art 5

**(11.)** *A2 nr. flagellum arts* (0) <9; (1) 10–15; (2) >15

**(12.)** *Lateral cephalic lobes* (0) rounded; (1) subacute, blunt

**(13.)** *Eyes* (0) medium; (1) small to absent; (2) large

Mouthparts

**(14.)** *Mandible palp art 3* (0) ≥ half art 2; (1) < half 2 or lacking

**(15.)** *Mandible palp art 2* (0) ≥3 setae; (1) 3–1 setae; (2) lacking

**(16.)** *Mandible palp art 3* (0) ≥2 setae; (1) one distally; (2) lacking

**(17.)** *Maxilliped outer plate : merus* (0) ≥ 0.5; (1) 0.5–0.2; (2) <0.2

Coxal plates

**(18.)** *Ratio length Cx2 : Cx1* (0) <2; (1) 2–2.5; (2) 2.5–3; (3) >3

**(19.)** *Cx2 ratio length : breadth* (0); ≥1.5; (1) < 1.5

**(20.)** *Cx4 ratio length : breadth* (0) l>b; (1) l=b; (2) l<b

**(21.)** *Cx4 distally excavated* (0) no; (1) yes

Gnathopods

**(22.)** *Gnathopod 1 dactylus* (0) ordinary; (1) spoon-shaped

**(23.)** *Gn 1 palm* (0) < half propodus length; (1) ≥ half propodus

**(24.)** *Gn 1 palm angle about* (0) no one = 180°; (1) blunt = 180–150° ; (2) acute = 120°; (3) transverse = 90°

**(25.)** *Gn1, 2 propodus shape* (0) similar; (1) different

**(26.)** *Gn 1 propodus shape* (0) rounded; (1) linear-rectangular; (2) triangular

**(27.)** *Gn1 carpus* (0) short, length< 2 breadth; (1) l=2b; (2) l<2b

**(28.)** *Gn1 merus* (0) short; (1) elongate; (2) freeprojecting

**(29.)** *Gn1 ratio carpus : propodus* (0) < prop.; (1) = prop.; (2) > prop.

**(30.)** *Ratio length propodus Gn1 : Gn2* (0) ≥0.75; (1) 0.75–0.33; (2) <0.33

**(31.)** *Gn2 ratio propodus : coxa male* (0) ≥1; (1) 1–0.66; (2) <0.66

**(32.)** *Gn2 ratio propodus:basis male* (0) ≥1; (1) 1–0.66; (2) <0.66

**(33.)** *Gn2 ratio propodus:basis female* (0) ) ≥1; (1) 1–0.66; (2) <0.66

**(34.)** *Gn2 ratio propodus : coxa female* (0) ≥1; (1) 1–0.66; (2) <0.66

**(35.)** *Gn2 palm male* (0) smooth; (1) toothed-serrated; (2) incision(s)

**(36.)** *Gn2 palm female* (0) smooth; (1) toothed-serrated; (2) incision(s)

**(37.)** *Gn2 carpus shape* (0) short, l<b; (1) ; elongate, l≥b

**(38.)** *Gn2 merus shape* (0) not lobate; (1) lobate

Peraeopods

**(39.)** *P4 ratio posterior margin of merus : propodus* (0) ≤1.33; (1) >1.33

**(40.)** *P5 basis* (0) ovoid widened; (1) rectangularly widened; (2) slim like basis P4

**(41.)** *P5 ratio anterior margin of merus : propodus* (0) ≤1.25; (1) >1.25

**(42.)** *P5 merus tip reaching* (0) no carpus; (1) 0.25–0.75 carpus; (2) full carpus

**(43.)** *P5 basis posterodistally* (0) no lobe; (1) small lobe; (2) medium lobe; (3) lobe wide and deep, reaching merus

**(44.)** *P5 basis width ratio maximum : minimum* (0); 1–1.1 (1) 1.1–1.4; (2) 1.4–1.6; (3) 1.6–1.8; (4) >1.8

**(45.)** *P6 basis* (0) ovoid widened; (1) narrow like P5; (2) rectangularly widened

**(46.)** *P6 basis hindmargin* (0); harmonically rounded (1) straight

**(47.)** *P6 basis posterodistal corner* (0) rounded lobe; (1) no lobe

**(48.)** *P6 merus length anterior: posterior margin* (0) =1; (1) >1

**(49.)** *P7 basis shape* (0) rounded; (1) narrow like P5; (2) rectangularly widened

**(50.)** *P7 merus reaching* (0) no carpus; (1) <0.5 carpus; (2) >0.5 carpus; (3) all carpus

**(51.)** *P7 ratio dactylus: propodus* (0) <0.5; (1) ≥0.5

**(52.)** *P7 basis posterior margin* (0); convex; (1) concave; (2) straight *Epimeral plates, Uropods + Telson*

**(53.)** *Ep3 posterodistally* (0) rectangular corner; (1) acute, 60–70°

**(54.)** *U1 rami* (0) equal; (1) very different

**(55.)** *U1 ratio peduncle : longer ramus* (0) ≤1; (1) 1–1.33; (2) >1.33

**(56.)** *U2 ratio of rami* (0) > 66%; (1) ≤ 66%;

**(57.)** *U2 ratio peduncle : longer ramus* (0) < 1; (1) > 1

**(58.)** *U2 peduncle spination* (0) no to weak; (1) strong

**(59.)** *U3 ramus ratio art 1 : art 2* (0) <1; (1) =1; (2) 1.1–1.9; (3) >2

**(60.)** *U3 ramus spination* (0) no; (1) 1–3 spines; (2) many

**(61.)** *Telson* (0); *length : breadth* ≤2 (1) ; *length : breadth* >2 (2) 3-dimensional

Schiecke (1973: 80 ff.) published in his doctoral thesis many suggestions about the morphological characters and their states in stenothoids in general. He surmises that the enlarged coxal plates are an advantage in a very densely structured environment such as cylindrical ramified branches in hydroids, bryozoans or algae, allowing stenothoids to “ride” on a branch gripping it with the paired anterior peraeopods from one side and with the posterior peraeopods from the other one, and hiding eggs or juveniles as well as the (usually) very thin posterior legs and uropods. Many species are known as associates with hydroids or bryozoans where they are observed to “steal” the little crustaceans collected and already paralyzed by the host from the tentacle-crown. But of course with stronger enlargement of Cx4 the vagility gets diminished and species with very large coxae are certainly very bad swimmers and probably detritus feeders, However, many stenothoids are excellent swimmers with an enlarged propodus on Gn2 and often with a long basis which affords great mobility, while Gn1 (inclusively Cx1) is extremely small.

It is remarkable that all mouthparts are always unusually long and narrow and it could be imagined that they all together function as a sucking device (it is said that the name steno-thoids stems from the narrow mouthparts, stenos meaning narrow in Greek). Mxp has reduced plates and Md has more or less reduced molars and palps, while pars incisivus and lacinia mobilis are very well developed with acute and robust “teeth”; also both maxillae have robust setae, which could help to divide the food parts already cut by the mandible.

In many stenothoids P3,4 are longer but weaker than P5–7, and all are kept parallel to the coxae and never twisted. Interesting are the quite often acutely lengthened meri (in P3,4 anteriorly, in P5–7 posteriorly) which warrant an additional capacity against fallling off the substrate.

## Taxonomy

### Genus Proboloides

Della Valle

Proboloides  Della Valle, 1893: 907

#### Type species.

*Metopa gregaria* Sars, 1882: 93, t. 4, fig. 6

*Proboloides* mainly occurs in the Atlantic, but nominal *Proboloides* species have been reported also from the Pacific, Indian and Antarctic oceans. Its species are often found living in deep waters and show a clear sexual dimorphism, usually their gnathopods are quite different in size and shape, often with a strongly incised Gn2 palm, with palmar corner well defined in females, but not defined in males, and robust peraeopods.

#### Diagnostic characters.

A1 peduncle art 1 usually short, length < 3× width, subequal to cephalon; A1 usually shorter than 2/3 body length, A1 accessory flagellum lacking. Md palp with a very short or lacking art3, poorly setose; Mx1 palp 2 arts; Mx2 inner plate ordinary; Mxp inner plates well separated, outer plates usually reduced (less than 0.2 of merus length). Ratio Cx2:Cx1 > 3. Cx2 length equal or more than 1.5 x the width. Gn1, 2 different in size and shape; Gn1 small, almost simple, rarely subchelate; carpus length equal to propodus; length of propodus Gn1 about half or less than half length of propodus Gn2; Gn2 palm has serrations or teeth, usually no incisions; Gn2 propodus is in males often, in females always smaller than Cx2; carpus shorter than wide, merus elongate. P5 basis linear, without posterodistal lobe; merus anterior margin shorter than 1.25 length of propodus anterior margin. P6, 7 basis expanded and lobate, merus tip reaching half to full length of carpus. Ep3 with acute posterodistal corner. U1 peduncle is longer than longer ramus. T length is shorter to equal the double width, triangular, laminar.

At the beginning of this study 16 species were known:

11 from the Atlantic, Pacific and Indian Ocean: *Proboloides anophthalmus* Ledoyer, 1986, *Proboloides calcaratus* (Sars, 1882), *Proboloides clypeatus* (Stimpson, 1853), *Proboloides grandimanus* (Bonnier, 1896), *Proboloides gregarius* (Sars, 1895), *Proboloides holmesi* Bousfield, 1973, *Proboloides pacificus* (Holmes, 1908), *Proboloides schokalskii* Gurjanova, 1946, *Proboloides schuleikini* Gurjanova, 1946, *Proboloides tundus* Barnard, 1962, *Proboloides zubovi* Gurjanva, 1951.

5 members from Antarctic-Subantarctic region: *Proboloides porcellanus* KH Barnard, 1932, *Proboloides rotundus* (Stebbing, 1917), *Proboloides stephenseni* Ruffo 1949, *Proboloides typicamimus* Andres, 1995, *Proboloides typicus* (Walker, 1906).

The differences between the current diagnoses of *Metopoides*, *Proboloides*, *Scaphodactylus* and *Torometopa* are still quite small and not satisfactory:

*Metopoides*. Mouthparts ordinary. Long antennae with 2-articulate flag. acc.; unspecialized gnathopods; short coxal plates; basis P6, 7 with weakly lengthened merus.

*Proboloides*. Md palp may have a shortened third article, the inner plates of Mxp may be fused. Antennae robust with 0–1 articulate acc. flag.; gnathopods with sexual dimorphism, Gn1 much smaller than Gn2; coxal plates enlarged; basis P6, 7 with strongly lengthened and widened merus.

*Scaphodactylus*. Mouthparts ordinary. Antennae with 2- articulate acc. flag.; gnathopods without or with sexual dimorphism (there are two groups within the genus); Gn1 dactylus spoon-shaped excavated; coxal plates small; basis P5 rectolinear with posterodistal lobe lengthened and widened; P6, 7 merus very weakly lengthened and widened.

*Torometopa*. Mouthparts ordinary. Antennae with 0–2- articulate acc. flag.; gnathopods without or with sexual dimorphism; coxal plates small or large; basis P5 rectolinear with posterodistal lobe lengthened and widened to varying degrees; P6, 7 merus weakly to strongly lengthened and widened. In short, characters of *Metopoides* and *Proboloides* together, but P5 basis with posterodistal lobe, which might have evolved independently. Thus this genus was the least convincing one.

To fill the gaps with question marks in the first matrix (see also Krapp-Schickel 2009), I studied the following species in detail:

### 1) Proboloides porcellanus KH Barnard, 1932: 111–112, fig. 61

This species has a small posterodistal lobe on P5 basis (like *Torometopa* and different from all other *Proboloides*), but P6, 7 bases are not rounded (like in all *Torometopa*), but narrowing distad. Gn1, 2 show a striking similarity with those of *Mesometopa sinuata* Shoemaker, 1964 (female) from the American west coast, but P5–7 in that species are totally different, and the other *Mesometopa* species are Pacific arctic-boreal (southernmost locality is S. California).

This species thus fits in no existing genus, and a new one is erected:

#### Malvinometopa gen. n.

urn:lsid:zoobank.org:act:17A26AB9-5D20-46FE-B5BA-4075DF82B8BD

##### Type species.

*Proboloides porcellanus* KH Barnard, 1932

##### Diagnostic characters.

Md palp with 3 arts, Mxp IP separated; Mx1, 2 unknown; A1, 2 peduncle strong, flagellum reduced to 4–6 arts. Cx4 not much wider than Cx2+3. P5 basis rectolinear, with small posterodistal lobe. P6, 7 basis narrowing distad, both with posterodistal small lobe reaching along half of ischium.

##### Etymology.

The type species was collected from the pharynx of a large ascidian in the Falkland Islands, in Spanish Islas Malvinas.

##### Remarks.

This genus has a posterodistal lobe on P5 basis like the members of *Torometopa*; itis different from all other known stenothoid genera by the rectangularly widened, distally narrowing basis of P6, 7. In *Metopella* and *Mesoproboloides* P5 is rectolinear without a posterodistal lobe, P6, 7 are differently widened; in *Hardametopa* P5–7 all have a slender basis. The genera *Metopelloides*, *Stenothoides*, *Vonimetopa* and *Zaikometopa* differ in having a 1-articulate Md palp, while the palp is absent in *Parametopella*.

##### Malvinometopa porcellana

(KH Barnard, 1932)

urn:lsid:zoobank.org:act:5DA70786-2BC5-415E-9953-E2FA6850218F

Figs 1 2

Proboloides porcellanus  KH Barnard 1932: 111–112, fig. 61
Torometopa porcellana Barnard and Karaman 1991: 700

###### Material examined:

Type material BMNH.

###### Redescription of type material:

####### Body

smooth. Eyes rounded, large.

####### Length:

5–9 mm.

####### Antennae.

A1 less than half of body length, peduncle robust, art 1 length about three times the width; acc. flag. absent, flagellum 6–7 arts. A2 subequal in length to A1, peduncle robust, art 4 < art 5, flagellum about as long as or shorter than peduncle art 5, with 7 arts.

####### Mouthparts.

Md palp with 3 arts, art 1 and 2 unusually long, art 3 much shorter than 1/3 length of art 2, with many distal setae. Mx 1, 2 unknown; Mxp IP not fused, 2/3 length of ischium; OP vanishing; dactylus long, subequal to propodus.

####### Coxae.

Cx2 with rounded anterior margin, straight behind, angle rounded without tooth; Cx3 trapezoid-shaped, distally widening, Cx4 not excavated, anterior and posterior margin rounded, about as long as wide.

####### Gnathopods.

Gn1, 2 propodi similar in shape, different in size. Gn1 dactylus ordinary; propodus with parallel margins, palm not defined, about twice as long as wide; carpus longer than propodus, subtriangular, longer than wide, proximally wider than distally; merus incipiently chelate; all articles densely beset with setae. Gn2 length of propodus more than 2/3 of Cx2; propodus subelliptical, twice the size of propodus Gn1; hind margin subequal to length of palm which has shallow incisions, palmar corner well defined by small tooth-shaped prolongation but no U-shaped incision. Dactylus same length as palm. Gn2 carpus shorter than wide, cup-shaped, merus not lobate.

####### Peraeopods.

P4 merus anterodistal margin somewhat lengthened. P5 dactylus half length of slim propodus; merus posterodistal margin not reaching half of carpus length, basis rectolinear with short posterodistal lobe. P6 basis hind margin straight, with posterodistal lobe similar to P5, merus posterodistal corner acutely lengthened and somewhat widened, not reaching to half of carpus length. P7 basis proximally widened with lobe, distad narrowing with small posterodistal lobe, hind margin crenulate and excavated; merus lengthened and widened, reaching about half carpus length.

####### Uropods.

U1 peduncle with many short robust setae, nearly twice as long as subequal rami; U2 peduncle also beset with many small robust setae, longer than longer ramus, rami somewhat unequal; U3 totally unarmed, peduncle longer than ramus, art 1 of ramus longer than art 2.

####### Telson.

Not reaching end of peduncle U3; less than twice as long as wide; distally tongue-shaped rounded, naked.

###### Sexual differences.

Small.

###### Distribution.

Falkland Islands.

###### Ecology.

From pharynx of a large ascidian.

###### Remarks.

*Malvinometopa porcellanus* has extremely shortened A1, 2, no Mxp OP, a stout Gn1 and aberrant P6, 7: is this an adaptation to the life inside the pharynx of ascidians, where they certainly cannot swim but only crawl? All we know is that space there is at a premium.

**Figure 1.**
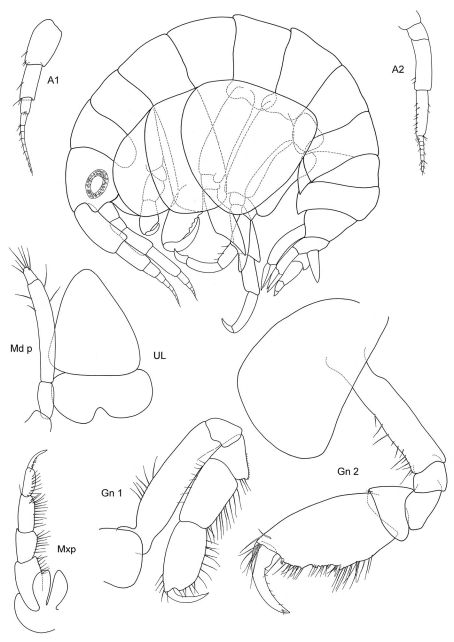
*Malvinometopa porcellana* (K.H. Barnard, 1932): Discovery Reports St. 51, Falklands.

**Figure 2.**
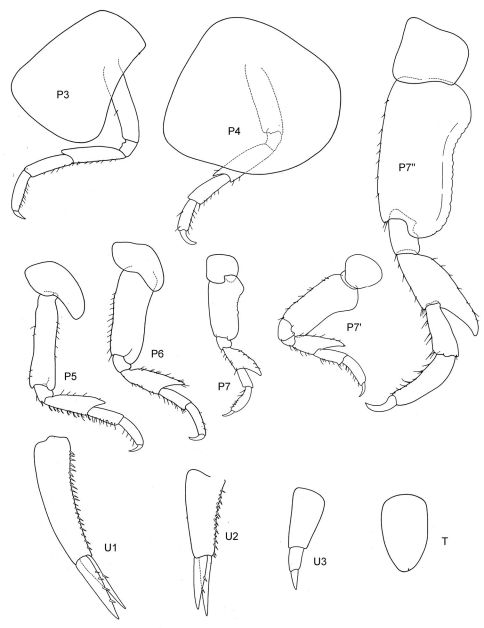
*Malvinometopa porcellana* (K.H. Barnard, 1932): Discovery Reports St. 51, Falklands.

### 2) Proboloides typicus (Walker)

Also this species is very sparsely described and figured. I found material at the Australian Museum Sydney, and compared it with one specimen deposited at the Verona Museum by Bellan-Santini. Both fit the written description by Schellenberg well, and this species clearly belongs in *Metopoides*:

#### Metopoides typicus

(Walker, 1906)

Figs 3 4

Proboliella typica Walker 1906: 14; 1907: 20–21 t.6 fig. 10
Proboloides typica Schellenberg 1926: 323–24 fig, 41; De Broyer et al. 2007: 213 not *Proboloides typica* KH. Barnard 1932: 109, f. 57
Metopoides  sp. Bellan-Santini and Ledoyer 1974: 700 fig. 38 B

##### Material examined:

Cape Bird, EBS, C3-C4, 70–100m, 14.12. 1971, several spec.; tide crack, near Cape Spencer, White Island, Ross Ice Shelf, 78°01'0"S, 167°20'0"E, 28.XII. 1976, coll. P. Ensor (AMS P 25504); Southern Rookery, Cape Bird, Ross Island, Antarctica (approx. 77°13'0"S, 166°27'0"E) AM P.80875 (1 slide); slide of “*Metopoides* sp. ”, Kerguelen,MNVCR.

##### Redescription after material from the Australian and Verona Museum:

###### Body

smooth. Eyes rounded, medium size.

###### Length.

3–3,5 mm.

###### Antennae.

A1 less than 2/3 of body length, peduncle robust, art 1 shorter than three times wide; acc. flag. with 2 (very small) articles, flagellum 10 arts. A2 subequal in length to A1, peduncle robust, art 4 somewhat > art 5, flagellum about as long as peduncle art 5, with 7 arts (Walker: without acc. flag., A1 reaching to the middle of the flagellum of A2).

###### Mouthparts.

Md incisor and raker spine row well developed; no clear molar cusp; palp with 3 arts, art 3 about 1/3–1/2 length of art 2, with 3 distal long setae (Walker: Md palp lacking third art, therefore creating a new genus *Proboliella*; but Schellenberg already noticed 1926: 323 fig. 41, that there is a well-developed third article). Mx 1 IP with 1 distal seta, OP with 6 strong robust setae, palp with 2 arts; Mx 2 inner plate ordinary, shorter than outer; Mxp IP not fused, 2/3 length of ischium; OP narrow, well developed, reaching more than half of merus length; dactylus long, subequal to propodus.

###### Coxae.

Cx2 with rounded anterior margin straight behind, angle rounded with small tooth; Cx3 narrow with parallel margins, Cx4 not excavated, inferior and posterior margin rounded, about as long as wide.

###### Gnathopods.

Gn1, 2 propodi different in size and shape. Gn1 dactylus ordinary; propodus with parallel margins, palm well defined (corner about 120°), somewhat longer than half length of propodus, about twice as long as wide; carpus shorter than propodus, triangular, longer than wide, merus incipiently chelate. Gn2 length of propodus more than 2/3 of basis in male, less in female; propodus subelliptical, twice the size of propodus Gn1; hind margin half length of palm which is in male and female with incisions, palmar corner well defined by acute tooth-shaped prolongation and U-shaped incision. Dactylus clearly shorter than palm, probably working together with robust setae of palmar corner. Gn2 carpus shorter than wide, cup-shaped, merus not lobate.

###### Peraeopods.

P4 merus anterodistal margin somewhat lengthened. P5 dactylus long, weak, much longer than half of slim propodus; merus posterodistal margin not reaching half of carpus length, basis slender without lobe. P6 basis hind margin harmonically rounded, clearly longer than wide, merus posterodistal corner acutely lengthened but not widened, not reaching to half of carpus length. P7 basis and merus similar to P6.

###### Epimeral plates.

Ep3 posterodistally lengthened to triangular corner.

###### Uropods.

U1 peduncle slightly longer than subequal rami, with many robust setae; U2 peduncle longer than shorter ramus, rami clearly unequal; U3 peduncle shorter than ramus, first article of ramus shorter than peduncle, ramus art 2 about ¾ of art 1.

###### Telson.

Not reaching end of peduncle U3; about twice as long as wide; distally triangulary pointed, medio-laterally with 2–3 robust setae.

##### Sexual differences.

Probably small.

##### Distribution.

Antarctica, Hut Point near Mc Murdo, 77.47°S (Walker 1906, 1907); S-Victoria Land, Gauß Station (Schellenberg 1926); White Island, Ross Ice Shelf, 78°01'0"S, 167°20'0"E, (AMS P 25504); Cape Bird, Ross Island, Southern Rookery, 77°13'0"S, 166°28'0"E (AMS P 80875).

##### Ecology.

Steeply sloping rock bottom with encrusted bryozoans and hydroids.

##### Remarks:

As this species clearly has an accessory flagellum (although tiny), unspecialized gnathopod propodi and neither much lengthened nor widened merus on P5–7, it has to be placed in the genus *Metopoides*, and even is a very “typical” representative of that genus.

**Figure 3.**
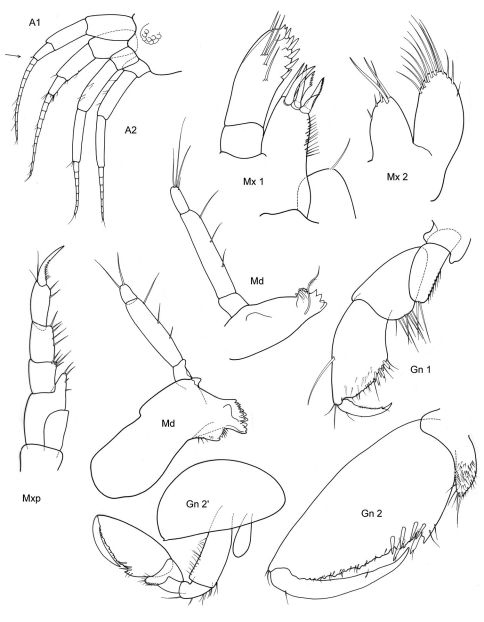
*Metopoides typicus* (Walker, 1906): Cape Bird, Southern Rookery; AMS.

**Figure 4.**
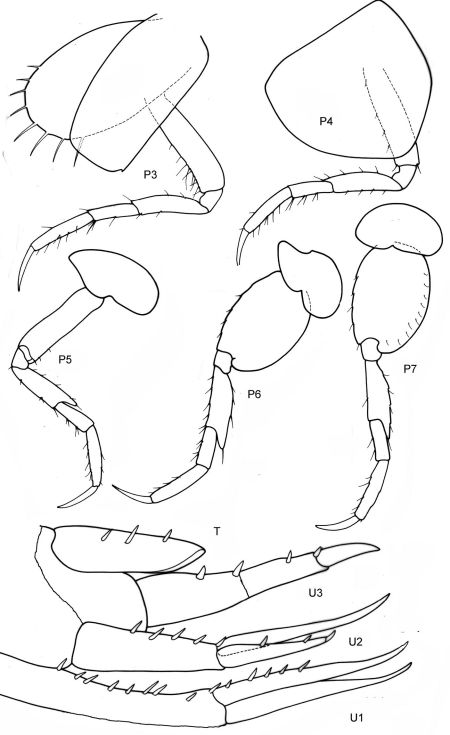
*Metopoides typicus* (Walker, 1906): Cape Bird, Southern Rookery; AMS.

### 3) Proboloides stephenseni Ruffo, 1949

Shortly after the war the possibility to check foreign literature was restricted. As only few characters were illustrated, the character states included in the matrix most probably were not always appropriate.

Until type material of *Metopa rotunda* can be checked, this species is synonymized with question mark to Stebbing’s species *“Metopa rotunda”*. In any case it should belong to *Metopoides* for many plesiomorphic character states.

#### Metopoides rotundus

(Stebbing, 1917)

Fig. 5

Metopa rotunda  Stebbing, 1917: 39–40, pl. XCVIB; K.H. Barnard 1940: 444; Griffiths 1974: 326
? Proboloides stephenseni  Ruffo, 1949: 15, fig. 1 (12–18), fig. 2 (1–5), fig. 3 (1)
? Torometopa stephenseni  Barnard & Karaman, 1991: 700
Proboloides rotundus  Barnard & Karaman, 1991: 696

##### Material examined.

Type material of *Proboloides stephenseni* MNVCr.

##### Redescription:

###### Body

smooth. Eyes rounded, large.

###### Length.

Male 3,5 mm.

###### Antennae.

A1 = A2, A1 less than half of body length, peduncle robust, art 1 length about three times the width; acc. flag. with 2 arts, flagellum 12 arts. A2 subequal in length to A1, peduncle robust, art 4 ≥ art 5, flagellum about as long as peduncle art 5, with 9 arts.

###### Mouthparts.

Md palp with 3 arts, art 1 and art 3 subequal, art 3 longer than 1/3 art 2, with 2 long distal setae. Mx 1 palp with 2 arts. Mxp IP separated, OP longer than half ischium; dactylus as long as propodus.

###### Coxae.

Cx2 with rounded anterior margin, straight posterior one, angle rounded with small tooth; Cx3 with parallel margins, Cx4 not excavated, anterior and posterior margin rounded, about as long as wide.

###### Gnathopods.

Gn1, 2 propodi similar in shape, different in size. Gn1 dactylus ordinary; propodus triangular, palm well defined, about twice as long as wide, about as long as hind margin; carpus shorter than propodus, trapezoid, longer than wide, with parallel margins; merus with very short distal free margin. Gn2 length of propodus = Cx2; propodus more than twice the size of propodus Gn1; hind margin much shorter than length of palm which has shallow incisions and crenulations, palmar corner well defined by small tooth-shaped prolongation but no U-shaped incision. Dactylus shorter than palm. Gn2 carpus shorter than wide, cup-shaped, merus not lobate.

###### Peraeopods.

P5 dactylus half length of slim propodus; merus posterodistal margin not reaching half of carpus length, basis rectolinear, width proximally and distally subequal, posterodistally rounded, but not lobed. P6, 7 similar, basis hind margin rounded, merus posterodistal corner shortly lengthened and somewhat widened, not reaching to half of carpus length.

###### Epimeral plates.

Ep3 posterodistal corner rectangular, but rounded.

###### Uropods.

U3 peduncle shorter than ramus, art 1 of ramus longer than art 2; peduncle with one short robust seta distally, ramus art 1 with 2 robust setae.

###### Telson

not reaching end of peduncle U3; less than twice as long as wide; distally pointed, marginally two robust setae.

##### Sexual differences.

Females unknown.

##### Distribution.

Antarctica, 70°23'0"S, 82°47'0"W (*Proboloides stephenseni* Ruffo, 1949). South Africa (*Proboloides rotunda* Stebbing, 1917).

##### Depth.

42 fathoms = 76,8 m (*Proboloides rotunda*, Stebbing 1917: 40)

After Barnard and Karaman (1991: 696) the genus *Proboloides* has its distribution in the Atlantic Ocean, S-Africa and the Antarctica. Therefore it was important to check also non-Antarctic species.

**Figure 5.**
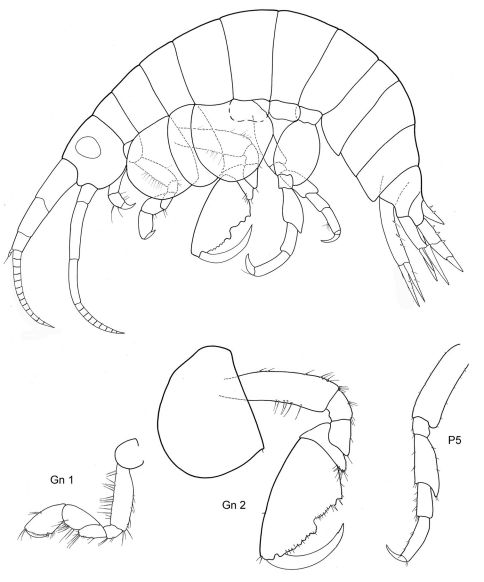
*Proboloides stephenseni* Ruffo, 1949: Antarctica (70°23'0"S, 82°47'0"W); MCV.

### 4) ? Proboloides holmesi Bousfield, 1973

#### ? Proboloides holmesi

Bousfield, 1973

Figs 6 7

Proboloides holmesi  Bousfield, 1973: 89, fig. 16 (2)

##### Remarks

Although the shape of the gnathopods (especially the simple Gn1) creates doubt if it could not belong in *Metopa* or *Stenula*, checking of the mouthparts showed at least that this species has a Md palp with 3 arts and a palp of Mx1 with ? 2 arts (although the articulation is not clear, see Bousfield 1973: 98). It is different from the other Atlantic members, but for the time being it should remain in the genus *Proboloides*.

**Figure 6.**
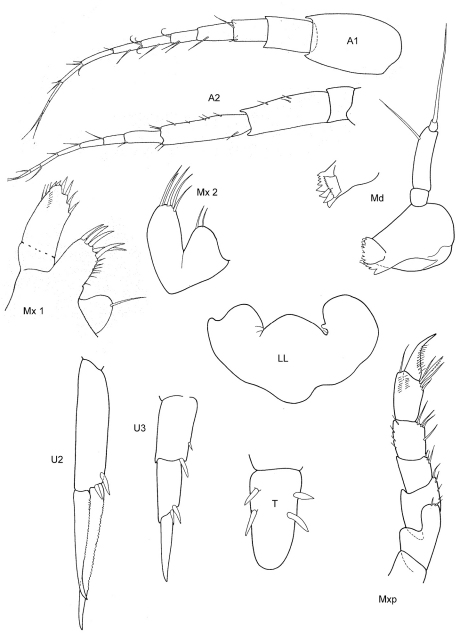
*?Proboloides holmesi* Bousfield, 1973: Raunefjorden near Bergen; MCV.

**Figure 7.**
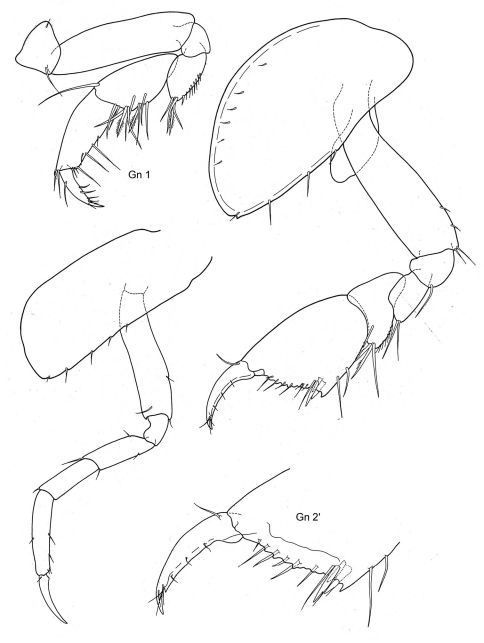
*?Proboloides holmesi* Bousfield, 1973: Raunefjorden near Bergen; MCV.

At the Verona Museum I found a tiny specimen called “*Metopa* sp.”(1,5 mm) which is extremely similar to *Proboloides holmesi*, except the rounded palmar corner (see Fig. 6, 7 and compare to Bousfield 1973 fig. 16 (2)); also here there seems to be a fine indistinct line in Mx1 palp. I am adding the illustration also to stress the fact how small the differences between the genera are, and to show that also the genus *Metopa* has to be included in this basic group of stenothoid genera.

##### Distribution.

S of Cape Cod; Vineyard Sound, Elizabeth Islands, Buzzard Bay (Bousfield 1973).

##### Ecology.

Mainly on sandy and shelly sand bottoms, among hydroids and bryozoans, in depth of 5–30 m (Bousfield 1973: 89).

### 5) Stenothoe aequicornis Stephensen, 1931

**Figure 8.**
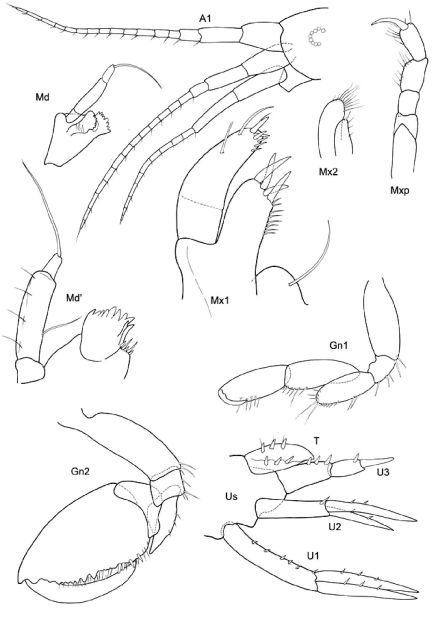
*Proboloides aequicornis* (Stephensen, 1931): after typical material from Atlantic, between Faroes and Iceland.

**Figure 9. F9:**
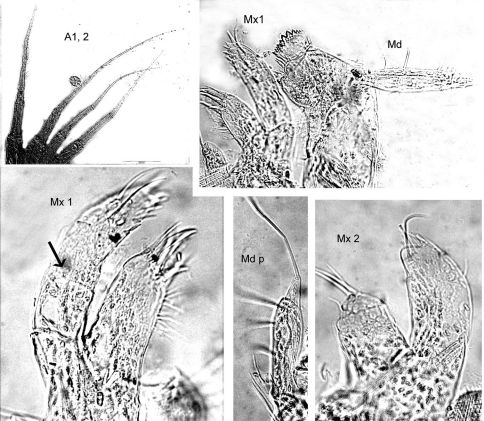
*Proboloides aequicornis* (Stephensen, 1931): as above, photographs of the material taken with Olympus BX51 with cell imaging software.

During a stay at the Copenhagen Museum I checked Stephensen’s type material of this species, as Barnard and Karaman (1991: 698) remarked “gnathopod 1 wrong, mouthparts unknown”. And they were right: there is no doubt about a clearly developed 3-articulated Md palp and therefore this species cannot be a member of *Stenothoe*, where there is no Md palp at all. Less clear is the structure of the palp of Mx1: as often in other specimens, the articulation is not easily seen (Fig. 8, 9). But this character should not be the only one deciding if a species belongs to *Metopa* or to *Proboloides*, and the shape of gnathopods brings this material into the vicinity of the latter.

#### Proboloides aequicornis

(Stephensen, 1931)

Figs 8 [Fig F9] 10

Stenothoe aequicornis  Stephensen, 1931: 198, fig. 59

##### Material examined.

Type material of *Stenothoe aequicornis* ZMUC.

##### Redescription.

###### Body

smooth. Eyes rounded, small.

###### Length.

Male 5 mm.

###### Head.

Lateral cephalic lobes bluntly angular.

###### Antennae.

A1 subequal A2 or A1 scarcely > A2. A1 peduncle robust, art 1 length about 2–3 x the width; art 3 only 1/3 of art 1 length; acc. flag. with 2 arts, flagellum about 1,5 x length of peduncle, 12–14 arts. A2 subequal in length to A1, peduncle subequal to flagellum, peduncle robust, art 4 ≥ art 5, flagellum with 9–11 arts.

###### Mouthparts.

Md palp with 3 arts, art 1 and art 3 subequal, art 3 longer than 1/3 art 2, with 1 long distal seta. Mx 1 palp with 2 arts (but articulation not easy to see, cf. fig. 8, 9). Mxp IP separated, OP longer very short; dactylus as long as propodus.

###### Coxae.

Cx2 with rounded anterior margin, straight or even somewhat concave behind, front angle rounded without tooth; Cx3 with trapezoid-shaped margins, Cx4 not excavated, anterior and posterior margin rounded, wider than long.

###### Gnathopods.

Gn1, 2 propodi different in size and shape. Gn1 dactylus ordinary; propodus elongate, about 3× as long as wide, palm well defined, much shorter than hind margin; carpus longer than propodus, triangular, nearly 3× longer than wide, with parallel margins; merus with very long distal free margin. Gn2 length of propodus > Cx2; propodus about 3× the size of propodus Gn1; hind margin much shorter than length of palm which has shallow incisions and crenulations, palmar corner scarcely defined by group of robust setae, no U-shaped incision. Dactylus subequal to palm. Gn2 carpus very short, cup-shaped, merus acutely lobate.

###### Peraeopods.

P5 dactylus > half length of slim propodus; merus posterodistal margin not reaching end of carpus length, basis rectolinear, width proximally and distally subequal, posterodistally rounded, but not lobed. P6 basis hind margin straight, merus posterodistal corner acutely lengthened and widened, reaching to end of carpus length; P7 similar to P6, but basis hind margin regularly rounded.

###### Epimeral plates.

Ep3 posterodistal corner acute, but rounded at the apex.

###### Uropods.

U3 peduncle shorter than ramus, art 1 of ramus subequal to art 2; peduncle with 3 robust setae distomarginally, ramus art 1 with 1 robust seta.

###### Telson.

Not reaching end of peduncle U3; less than twice as long as wide; distally pointed, marginally 3 robust setae.

**Figure 10.**
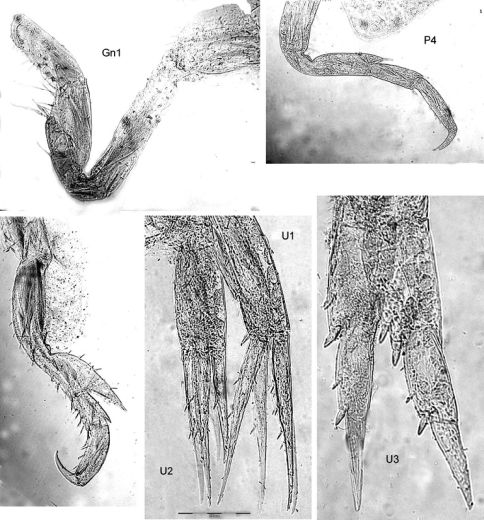
*Proboloides aequicornis* (Stephensen, 1931): as above, photographs of the material taken with Olympus BX51 with cell imaging software.

##### Sexual differences.

Females unknown.

##### Distribution.

Between Faroes and Iceland, 375 m depth.

#### 

At the Verona Museum I looked for the only species of the genus Torometopa cited in Barnard and Karaman (1991), where there is a question mark behind the genus name:

### 6) Proboloides armata Ledoyer, 1986: 966, fig. 381, 382 A

*Torometopa? armata* Barnard & Karaman, 1991: 700

It is the unique (female) type specimen from Îles Glorieuses N of Madagascar, from 3718 m depth.

Unfortunately I could not examine the slide and confirm the drawings of the sub-rectangularly widened Gn1 propodus and the 1-articulated Md palp, both very unusual characters in our treated group, as this type must be deposited elsewhere.

But at the Victoria Museum Melbourne I found a species from the Bass Strait from 770 m clearly belonging also to this basic species-complex of stenothoids, having a posterodistal lobe on P5 basis. To my big surprise it turned out that this species too had a 1-articulate Md palp.

As this character- combination does not fit any of the extant stenothoid genera, a new one was erected:

#### Victometopa gen. n.

urn:lsid:zoobank.org:act:29F3DCFA-418C-4865-BA45-98F22273BFB8

##### Type species.

*Victometopa rorida* sp. n.

##### Probably also included:

*Victometopa armata* (Ledoyer, 1986), comb. n.

##### Diagnostic characters.

Md palp with 1 art, Mxp OP reduced. P5 basis rectolinear, with posterodistal lobe. P6, 7 basis widening.

##### Etymology.

The stem -metopa combined with the first letters of “Victoria”, for expressing admiration for the rich collection at the Victoria Museum Melbourne (Australia).

##### Victometopa rorida sp. n.

urn:lsid:zoobank.org:act:

Figs 11 12 

###### Holotype.

Male 4.4mm. Cruise 79-K-1, Stn 34, 30°38'42"S, 148°49'24"E, Flinders Canyon, eastern Bass Strait, 770 m, 27.3.1979. Sediment: shell/sand, gear: dredge. MVM J 39597

###### Paratype.

Male 4.2mm. Same locality.

###### Etymology.

The Latin adjective *roridus* means “set with dew” and should stress the “pearls” on the Cx3 in this species.

###### Description.

####### Length.

4.2 - 4.4 mm

####### Body.

Smooth. P3–7 all clearly prehensile, with falcate-concave, strikingly long merus and strong dactylus opposing with spinose propodus.

####### Head.

Lateral cephalic lobes subacute or blunt, triangular. Eyes rounded, medium.

####### Antennae.

A1: body length ≥ 0.66 body, ped. art 1 l:b >3; ped. art 2 ≥art 1; art 1 =cephalon; ped. art 3 ≤0.3 art 1; acc. flag. absent; flagellum arts 11–20. A2 ped .art 5> flag., ped. art 4 =art 5, nr. flag. arts ≤ 9 (A2 broken into pieces, thus indications not totally sure).

####### Mouthparts.

Mdb palp one long article, on tip a fine articulation-line visible, marginally no setation, distally 1 long and 1 shorter seta. Mxp outer plate reduced.

####### Coxae.

Cx2:Cx1 ratio of length >3. Cx2 l:b (l=parallel post. margin) < 1.5. Cx3 unusually widened, nearly as long as wide, distoposterior margin with strong “pearls” or stridulation ridges. Cx4 l<b, distally not excavated.

####### Gnathopods.

Gn1 dactylus ordinary. Gn1 palm subequal to half propodus length; propodus palm angle 180–150°, blunt; propodus shape rounded, l≤ 2b; carpus l>2b; merus free projecting; carpus longer than propodus. Gn1 propodus < 0.33 Gn2 propodus. Gn1, 2 propodus shape different. Gn2 propodus ≥coxa and basis in male, palm in male smooth, only at dactylus-insertion some serrations; carpus very short, merus small, subquadrangular, not lobate.

####### Peraeopods.

In all dactylus clearly longer than propodus. P3,4 merus long, falcate curved, nearly twice the length of propodus. P5 basis distally somewhat widened but strongly lengthened to lobe maximal to minimal breadth 1.4–1.6; merus also nearly twice the length of propodus, posterodistal tip neither lengthened nor widened. P6 basis hind margin with straight margins, merus anterior and posterior margin subequal, distally not lengthened, reaching no carpus. P7 basis wider than in P6, but posterior margin also rather straight.

####### Epimeral plates.

Ep3 posterodistally rectangular corner.

####### Urosome.

U1 rami equal. U1 peduncle longer than ramus. U2 rami different, the shorter is longer than 0.66 % of the longer one, peduncle is longer than rami, spination weak. U3 with very long peduncle, much longer than ramus; ramus art 1:2 <1, spination poor.

####### Telson.

l:b ≤2, distally rounded, marginally with two strong robust setae.

**Figure 11.**
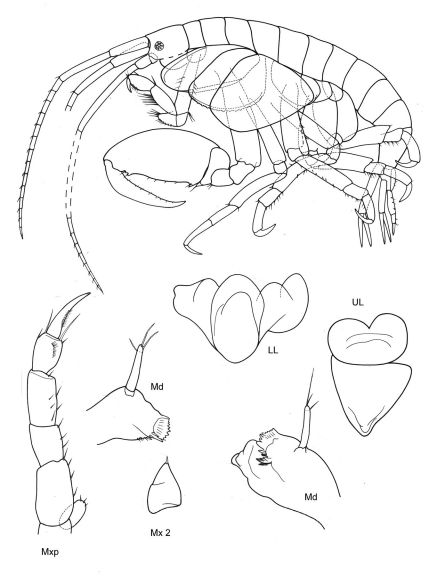
*Victometopa rorida* gen. n. sp. n.: Habitus ? male 4.4 mm; mouthparts UL, Mx1, 2 Md, LL, Mxp.

**Figure 12.**
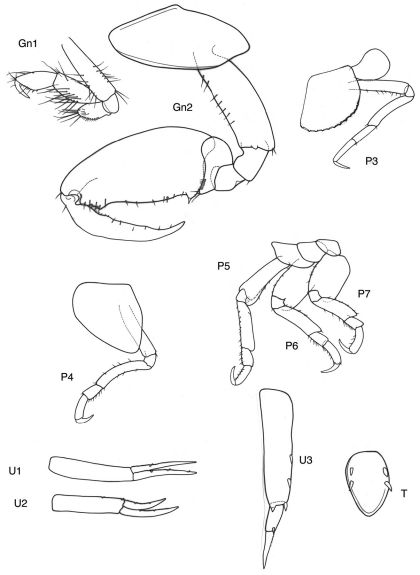
*Victometopa rorida* gen. n. sp. n.: Gn1, 2 = gnathopod 1, 2; P3-7 = peraeopods 3-7; U1-3 = uropods 1-3; T = telson.

## Cladistic analysis

Figure 13, 14.

**Figure 13.**
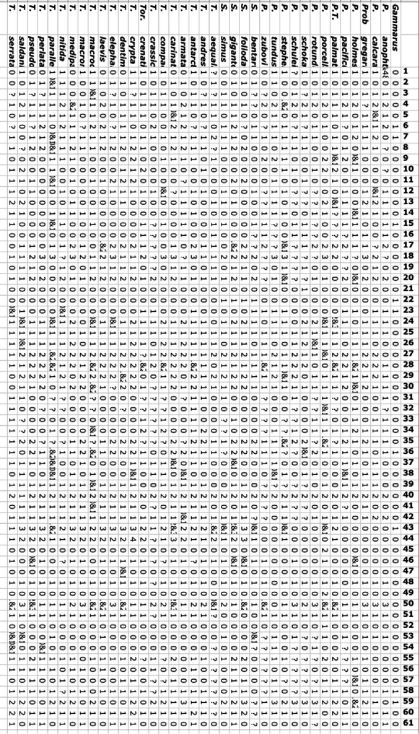
Matrix for 38 taxa and 61 characters (see description for character states in the text).

A matrix of 38 species and 61 characters was built (Fig. 13): all presently known species in the genera *Proboloides*, *Torometopa* and *Scaphodactylus* were included. A hypothetical *Gammarus* species was chosen as out-group (see also Krapp-Schickel 2009, Fig. 5 without the species of *Metopoides*).

**Figure 14.**
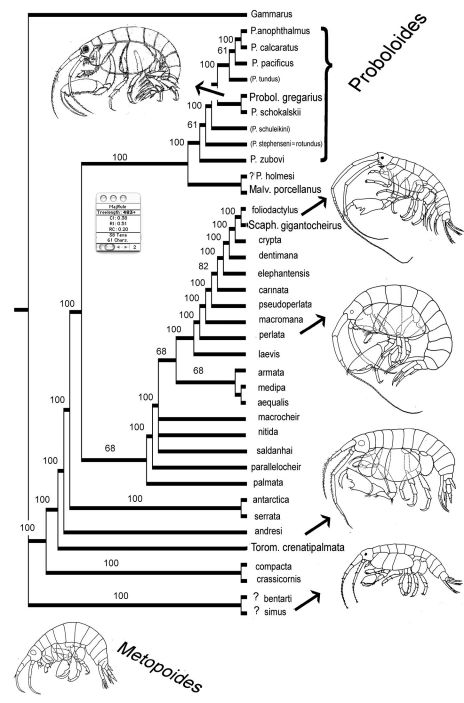
Heuristic analysis using 38 taxa and 61 characters: majority rule tree of 28 trees with length = 483. Genera abbreviated with more than one letter indicate type species; names written with small letters indicate that species are put in synonymy.

The programs MacClade 4.06 (Maddison and Maddison 2003) and PAUP 40B.10 (Swofford 2002) were applied. Using 38 taxa and 61 characters a heuristic analysis with a hypothetical *Gammarus* as an outgroup - species was performed and the majority rule consensus tree of 28 trees illustrated in Fig. 14. The difference between the trees concerned only the arrangement within the groupings.

Heuristic search settings:

Optimal criterion = parsimony

Characters were unweighted and unordered

Gaps are treated as “missing”

Multistate taxa interpreted as polymorphism

Starting tree(s) obtained via stepwise addition

Addition sequence: random

Number of replicates = 50

Starting seed = 1191736759

Number of trees held at each step during stepwise addition = 7

Branch-swapping algorithm: tree-bisection-reconnection (TBR)

Steppest descent option not in effect

Initial “MaxTrees” setting = 200 (will be auto-increased by 100)

Branches collapsed (creating polytomies) if maximum branch length is zero

‘MulTrees’ option in effect

Topological constraints not enforced

Trees are unrooted

Total number of rearrangements tried = 46863460

Score of best tree(s) found = 486

Number of trees retained = 28

Tree length = 483

CI = 0,38

RI = 0,51

RC = 0,20

## Results

At the beginning of the present analysis 16 species were cited for *Proboloides*:

Eight species were reported from the Atlantic or Arctic Ocean: *Proboloides calcaratus*, *Proboloides clypeatus*, *Proboloides grandimanus*, *Proboloides gregarius*, *Proboloides holmesi*, *Proboloides schokalskii*, *Proboloides schuleikini*, *Proboloides zubovi*. In most species at least the females have a pronounced palmar corner in Gn2, while Gn1 is weak and slender. In the deep-water species *Proboloides calcaratus* and *Proboloides gregarius* the males have Gn2 propodus + dactylus very much lengthened and the eyes large, Cx3 has no parallel margins, but becomes wider distally and is not much narrower than Cx 4, while *Proboloides holmesi* has a narrow Cx3 with parallel margins, very different from Cx4.

*Proboloides clypeatus* must remain a species dubia, as it is too poorly described.

The species *Proboloides gregarius* and *Proboloides schuleikini* (originally only subspecies of *Proboloides gregarius*) show differences only in the even more elongated Gn1 in the latter, and I think *Proboloides schuleikini* is a big female of *Proboloides gregarius*. I have also my doubts about the description of *Proboloides grandimana* (Bonnier), where all details match *Proboloides gregarius* except the big and triangular Cx1 which should be even larger than Cx2, an extremely unusual character in stenothoids; it seems quite probable that this is an error and that Cx2 is repeated. - Branch et al. (1991) illustrate a *Proboloides* sp. with U3 with 2 rami, which undoubtedly is also an error of the drawing.

Thus the only certain Atlantic-Arctic members are *Proboloides calcaratus*, *Proboloides gregarius*, *Proboloides schokalskii* and *Proboloides zubovi*.

There are 5 nominal *Proboloides* species from S-Africa and the Antarctic-Subantarctic region: *Proboloides porcellanus*, *Proboloides rotundus*, *Proboloides stephenseni*, *Proboloides typicamimus*, *Proboloides typicus*. The species *Proboloides stephenseni* and *Proboloides rotundus* are morphologically similar and may be synonymized; *Proboloides typicus* is redescribed and both could be transferred to *Metopoides*, as they have more plesiomorphic character states than members of *Proboloides*. - *Proboloides porcellanus* is redescribed and is the type of a new monotypic genus *Malvinometopa*.

The remaining species *Proboloides typicamimus* would then be the only member of the genus *Proboloides* living in the Antarctic, but it seems quite probable that also this species does not belong to this genus. But it is incompletely described though, based on a single specimen and knowledge about its character states is still very inadequate.

There are two nominal Pacific species of *Proboloides* remaining, *Proboloides tundus* and *pacificus*, which may well be synonymous: the shape of Gn2 matches (the only illustrated detail of the first), and the written description of the shape of P5-7 merus in *Proboloides tundus* “narrow, scarcely produced” matches the description by Shoemaker 1964 for *Proboloides pacificus* “very slightly expanded”. Furthermore both species were found off California at greater depth (302 fathoms = 552 m and 718 fathoms = 1313 m, among hydroids on the back of a spider crab). Shoemaker describes and illustrates *Proboloides pacificus* with slender P5–7 merus, but at the end of his remarks he adds: “as shown here, the merus [of the last peraeopods] is widely expanded”, which must be a lapsus linguae.

*Proboloides anophthalmus* is the only species living in the Indian Ocean (Madagascar). It is very similar to the Atlantic species *Proboloides? holmesi*, however there are some differences in the shape of T, the presence of pearls on the Cx3 margin and the shape of A1, 2. This deep-sea species has no eyes.

Two species can be added here:

“*Metopa nordmanni*” sensu Shoemaker 1955: 128 fig. 10 a-j (non *Metopa nordmanni* Stephensen) has to be described as new member of *Proboloides*, but as I could not see the material it must be cited as *Proboloides* sp. (Shoemaker, 1955) for the time being.

*Proboloides aequicornis* (Stephensen, 1931), as explained above.

After the thorough check of 16 species, eight (plus one doubtful member) remain belonging to *Proboloides* and show that the title in this series of papers is no longer appropriate, as none of them was found in the Austral-Antarctic region:

aequicornis (Stephensen, 1931: 198 fig. 59); between Faroes and Iceland, N-Atlantic.

anophthalmus Ledoyer, 1986: 965–66 (Madagascar, 335–450 m); Indian Ocean.

calcaratus (Sars, 1882: 92, t.4 sub *Metopa calcaratus*, 1895: 247, t. 85 sub *Probolium. c*.; 1992: 247 t. 85), Atlantic.

gregarius (Sars, 1882: 93; 1895: 245 pl. 84), Atlantic; = probably *grandimanus* (Bonnier 1896: 638 sub *Probolium grandimanum*), species dubia, Atlantic; = probably *schuleikini* Gurjanova 1946: 283 sub *Proboloides gregarius* ssp*. schuleikini*, Arctic.

**?** holmesi Bousfield 1973: 89 fig. 16/2, Atlantic.

pacificus (Holmes, 1908: 524), Pacific O, = ? *tundus* JL Barnard 1962: 147–149, Pacific.

schokalskii Gurjanova, 1946: 283, Arctic, Kara Sea

**sp.** Shoemaker 1955: 128, Arctic, Point Barrow

zubovi Gurjanova 1951: 412–13, Arctic, Kara Sea

Species dubia.

*Proboloides clypeatus* (Stimpson, 1853: 51 sub *Stenothoe clypeata*), Atlantic.

Species incertae sedis.

*? Metopoides typicamimus* Andres 1995: 355–364, Antarctica. [This species may very well belong to *Metopoides*, but has very elongate antennae and lacks OP on Mxp, or at least not seen with certainty, both advanced character states. More material is needed for solving this question].

*? Proboloides holmesi* Bousfield 1973: 89, fig. 16(2). [Aberrant member of this genus. May belong in *Metopa*].

Species transferred **to Metopoides.**

*Metopoides rotundus* (Stebbing, 1917) (= ? *Proboloides stephenseni* Ruffo, 1949)

*Metopoides typicus* (Walker, 1906)

## Key to 15 members of Metopoides species (including ?Metopoides typicamimus)

**Table:** 

1	P7 basis posterodistally regularly rounded	2
–	P7 basis distally clearly narrowing	13
2	Gn2 male palm with incisions, excavations and teeth; palmar corner about 90°	3
–	Gn2 male palm smooth or serrated	4
3	Gn1 carpus < propodus; P5–7 merus distoposteriorly lengthened, clearly reaching more than half length of carpus, in P7 even more than full carpus length; U3 ramus art 1 = art 2, peduncle and ramus art 1 with 1 robust seta each	*Metopoides pollex* Krapp-Schickel, 2008 (3–4 mm)
–	Gn1 carpus > propodus; P5–7 merus posterodistal tip not reaching half length of carpus; U3 ramus art 1 > art 2, peduncle and ramus art 1 with many robust setae	? *Metopoides typicamimus* Andres, 1995 (3–3,5 mm)
4	Gn1 carpus twice as long as wide; propodus widening distad, with concave palm and upturned palmar corner	*Metopoides clavatus* Schellenberg (5.5–8 mm)
–	Gn1 carpus not as long; propodus not as above	5
5	P6 basis widened, but anterior and posterior margin parallel, not convex	6
–	P6 basis posterior margin convex, rounded as P7	7
6	Gn1 short and wide, propodus and carpus l < 2b; Gn2 palm 1/3 of total length of propodus; U3 ramus art 1 subequal length of peduncle	*Metopoides sarsii* (Pfeffer) (2.8–6.5 mm?!)
–	Gn1 elongate, propodus and carpus l > 2b; Gn2 palm > 1/3 of total length of propodus; U3 ramus art 1 < length of peduncle	*Metopoides lanceolatus* Rauschert (3–4 mm)
7	Cx4 inferior margin distinctly excavated, concave	8
–	Cx4 inferior margin convex or only slightly excavate	9
8	Gn2 fem. propodus with parallel margins, palmar corner about 150°, width < half length of anterior margin	*Metopoides cf. heterostylis* (3 mm), *Metopoides heterostylis* Schellenberg (2.8–3.3 mm)
–	Gn2 fem. propodus widest at palmar corner, which is < 120°, width > half length of anterior margin	*Metopoides latus* Rauschert (2.8–3.4 mm)
9	Gn1, 2 propodus with parallel margins, shape very similar; P7 merus twice as wide as carpus, reaching half length of carpus	*Metopoides curvipes* Schellenberg(juv. fem. 2 mm)
–	Gn2 propodus widening	10
10	Gn2 crenulated, in the middle of the long palm a semicircular excavation	*Metopoides rotundus* (Stebbing, 1917), ?= *Metopoides stephenseni* (Ruffo, 1949) (3.5 mm)
–	Gn2 palm serrated or smooth	11
11	Gn2 palmar corner well defined by short and acute tooth as well as shallow excavation; P6, 7 merus distoposterior tip not reaching half length of carpus	*Metopoides typicus* (Walker, 1906) (3-3.5 mm)
–	Gn2 palmar corner smooth	12
12	U3 peduncle = ramus, strongly spinose; P7 basis posteriorly semicircularly rounded	*Metopoides bellansantiniae* (Bushueva) (3 mm)
–	U3 peduncle < ramus, naked; P7 basis oval	*Metopoides magellanicus* (Stebbing) (2.8–3 mm)
13	Gn2 propodus twice as long as wide; A1>A2	14
–	Gn2 propodus clearly much more than twice as long as wide; A1< A2	15
14	Gn1 length of propodus = carpus length; P7 basis proximally twice as wide as distally and about twice as long as the distal width	*Metopoides longicornis* Schellenberg (2–3 mm)
–	Gn1 length of propodus < carpus length; P7 basis proximally only a little wider than distally, about three times as long as the distal width	*Metopoides angustus* Rauschert (3.2 mm)
15	P6 basis elliptical, distally and proximally about the same width; P7 posterior margin regularly convex, but posterodistally no lobe	*Metopoides ellipticus* Schellenberg (4.5 mm)
–	P6, 7 basis trapezoid shaped, distally distinctly narrower than proximally	*Metopoides leptomanus* Rauschert (3.6–3.9 mm)

## Key to 9 members of Proboloides (including ? Proboloides holmesi and Proboloides sp.)

**Table:** 

1	Gn2 male palm identical to propodus length; propodus longer than 2× width	2
–	Gn2 male palm not identical to propodus length; propodus shorter than 2× width	3
2	Gn2 male propodus with short serrated margin near dactylus-insertion, then rectangular corner, two humps along the remaining palmar margin; Gn2 female palm with similar serrated part near dactylus insertion, remaining palmar margin somewhat excavated	*Proboloides gregarius* (Sars, 1882) (5–6 mm, N-Atlantic, Arctic)
–	Gn2 male propodus with short serrated margin near dactylus insertion, defined by blunt corner, remaining palmar margin smooth; Gn2 female propodus rounded, smooth, palm = hind margin	*Proboloides calcaratus* (Sars, 1882) (5–6 mm, N-Atlantic)
3	Gn2 male palmar margin not defined by acute tooth	4
–	Gn2 male palmar margin defined by acute tooth	5
4	Gn2 propodus l:b about 3:2, palm about the length of remaining hind margin, corner rounded; Gn1 simple, triangular carpus < propodus	*?Proboloides holmesi* Bousfield, 1973 (2,5 mm, Pacific)
–	Gn2 propodus l:b about 2:1, palm not defined, dactylus reaching along 2/3 of palm which is unregularly serrated; Gn1 subchelate, elongate carpus with parallel margins, > propodus	*Proboloides aequicornis* (Stephensen, 1931) 5 mm, Faroes, N-Atlantic
5	Gn2 male, female palm longer than remaining hind margin of propodus; Gn1 propodus triangular, palmar corner about 120°	*Proboloides schokalskii* Gurjanova (5 mm, Kara Sea)
–	Gn2 male, female palm not longer than remaining hind margin of propodus	6
6	Gn2 male next to dactylus insertion strongly serrate; besides the palm-defining strong and acute tooth in the middle of the palm another one, followed by a small narrow incision; in male, female P7 basis oval	*Proboloides pacificus* (Holmes, 1908) (6 mm, Pacific)
–	Gn2 palm not divided by additional tooth, regularly serrated from dactylus-insertion to corner	7
7	Gn1 carpus clearly longer than propodus, more than 4× longer than wide	*Proboloides* sp. (see “Proboloides nordmanni” in Shoemaker 1955 p. 30 fig. 10(a-j)
–	Gn1 carpus subequal to propodus	8
8	Gn1 propodus l:b = 2; P6 basis with regularly rounded margin, merus reaching end of carpus	*Proboloides anophthalmus* Ledoyer, 1986 (2,5 mm, Madagascar)
–	Gn1 propodus l> 2b; P6 with straight, parallel margins, merus not reaching end of carpus	*Proboloides zubovi* Gurjanova, 1951 (5 mm, Kara Sea)

The habitus sketches added to the resulting tree in Fig. 14 (from above: *Proboloides*, a large *Scaphodactylus*, *Torometopa* with A1> A2, *Torometopa* with A1 < A2, small *Scaphodactylus* and *Metopoides*) may give an idea about the differences in the body shapes in this basic group of stenothoids: e.g. members of *Proboloides* have a disto-posteriorly lengthened and widened merus on the last three peraeopods, which is much less the case in all other groups. Ed Bousfield (in litteris) opines that this must have an important hydrodynamic function, which could well be imagined. Also the relative length and width of Cx 4 (in *Scaphodactylus gigantocheirus* strikingly small) or the relation of the antennae (in the group near *Torometopa antarctica* and *Torometopa crenatipalmata* with the second one always being longer and stronger) could tell us something about the swimming (or even digging?) ability, if we would know more about their life style. However, it seems probable that *Proboloides* members are mainly free-living and do not live in association with, or at least not inside of, other animals; they ought therefore to be good swimmers and have a rather strong sexual dimorphism.

The remaining species in Fig. 14., not belonging to the genus *Proboloides*, will be treated in the following and final part.

## Supplementary Material

XML Treatment for Proboloides

XML Treatment for Malvinometopa

XML Treatment for Metopoides typicus

XML Treatment for Metopoides rotundus

XML Treatment for Proboloides holmesi

XML Treatment for Proboloides aequicornis

XML Treatment for Victometopa
